# Mechanism of cyanobacterial ferredoxin‐dependent flavin thioredoxin reductase reveals thiolate‐FAD charge transfer and catalytic asymmetry in a homodimer

**DOI:** 10.1111/febs.70210

**Published:** 2025-08-23

**Authors:** Martha Minjarez‐Saenz, Víctor Correa‐Pérez, Maribel Rivero, Alejandro Hernández‐Gómez, Marta Martínez‐Júlvez, Rubén M. Buey, Federico Gago, Monica Balsera, Milagros Medina

**Affiliations:** ^1^ Department of Biochemistry and Molecular and Cellular Biology, Faculty of Sciences and Institute for Biocomputation and Physics of Complex Systems (BIFI) University of Zaragoza Spain; ^2^ Department of Abiotic Stress Institute of Natural Resources and Agrobiology of Salamanca (IRNASA‐CSIC) Spain; ^3^ Metabolic Engineering Group, Department of Genetic and Microbiology, Faculty of Biology University of Salamanca Spain; ^4^ Department of Biomedical Sciences, School of Medicine and Health Sciences University of Alcalá Alcalá de Henares Spain

**Keywords:** conformational dynamics, disulfide, ferredoxin, flavoenzymes, homodimers, structural asymmetry, thioredoxin

## Abstract

Ferredoxin‐dependent flavin thioredoxin reductases (FFTRs) catalyze the reduction of the disulfide bond in thioredoxins using electrons transferred from ferredoxin, and therefore play a pivotal role in cellular disulfide relay reactions. FFTRs are essential in cyanobacteria such as *Gloeobacter* and *Prochlorococcus*, in which they serve as the sole thioredoxin reduction system, as well as in certain *Clostridium* species, where they are implicated in processes such as sporulation. Despite the well‐established role of ferredoxin in reducing FFTRs, the underlying mechanistic details remain poorly understood. This study examines the catalytic cycle of FFTR from *Gloeobacter violaceus*, focusing on the role of its redox‐active disulfide in electron transfer. We demonstrate that FFTR has a highly negative flavin adenine dinucleotide (FAD) midpoint reduction potential, which explains its preference for ferredoxin over nicotinamide adenine dinucleotide phosphate (NADPH) as an electron source. Spectroscopic detection of a thiolate–flavin charge transfer complex along the enzyme reduction pathway provides the first experimental evidence of a previously elusive FFTR catalytic conformation. Our results further reveal sequential FAD reduction within the enzyme homodimer that strongly suggests monomer asymmetry. Moreover, the impaired flavin reduction observed in an enzyme variant lacking the disulfide highlights the essential role of this redox group in efficient electron transfer. These findings deepen our understanding of FFTR's unique functional adaptations and evolutionary significance. More broadly, they provide a framework for exploring similar electron transfer mechanisms in other flavoproteins with a view to expanding our understanding of their redox biochemistry.

## Graphical abstract

Ferredoxin‐dependent flavin thioredoxin reductases (FFTRs) regenerate reduced thioredoxin, sustaining dithiol–disulfide exchange reactions that regulate protein activity in select organisms. In cyanobacterial FFTRs, we describe the formation of a thiolate–flavin charge transfer complex, asynchronous reduction of the two FAD cofactors within the homodimer, and coexistence of two catalytically competent conformations—flavin‐reducing (FR) and flavin‐oxidizing (FO). These mechanistic insights deepen our understanding of FFTRs, offering a framework applicable to other flavoenzymes to expand our understanding of their redox biochemistry.

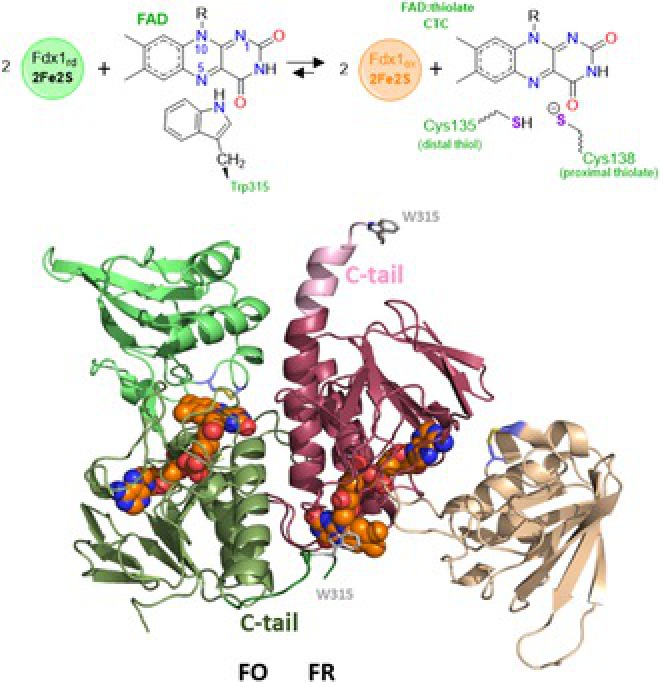

**Abbreviations**
CTC: charge transfer complex
dRF: 5‐deazariboflavin
DTH: dithionite
DTNB: 5,5′‐dithiobis(2‐nitrobenzoic acid)
ET: electron transfer
Fdx: ferredoxin
FFTR: ferredoxin‐dependent flavin thioredoxin reductase
FO: flavin‐oxidizing
FR: flavin‐reducing
HMW: high molecular weight
hq: hydroquinone
LMW: low molecular weight
NTR: NADPH‐dependent Thioredoxin Reductase
ox: oxidized
sq: semiquinone
Trx: thioredoxin

## Introduction

Cyanobacterial genera such as *Gloeobacter* and *Prochlorococcus* exhibit a distinctive redox system characterized by the absence of NADPH‐dependent Thioredoxin Reductases (NTRs) and iron–sulfur ferredoxin‐thioredoxin reductases (FTRs) [1, 2, 3]. Instead, they rely on ferredoxin‐dependent flavin thioredoxin reductase enzymes (FFTR) as their sole thioredoxin (Trx) reduction system [4]. FFTRs catalyze electron transfer (ET) from reduced ferredoxin (Fdx) to Trx, thus enabling downstream redox processes essential for cellular function [5]. Beyond cyanobacteria, FFTRs are also present in some *Clostridium* species, where they are implicated in processes like sporulation, a key adaptation for bacterial survival, dissemination, and fermentation [6, 7, 8]. Despite their evolutionary relationship to low‐molecular weight NTRs (LMW‐NTRs) [9], FFTRs display unique structural and functional features [4, 5]. LMW‐NTRs are homodimers, with each protomer consisting of two structural domains connected by two antiparallel β‐strands: the FAD‐binding domain, which accommodates a non‐covalently bound FAD, and the NADPH‐binding domain, which interacts with NADPH and contains a CxxC motif that mediates redox‐dependent reversible disulfide bond formation. Within each monomer, these domains undergo distinct conformational transitions between the flavin‐reducing (FR) and flavin‐oxidizing (FO) states during the catalytic cycle. In the FR conformation, the oxidized flavin cofactor is ready to accept electrons from NADPH, while the dithiol of the CxxC motif is solvent‐exposed, allowing interaction with Trx. Upon FAD reduction by NADPH and CxxC oxidation to a disulfide by Trx, the enzyme transitions to the FO conformation, where the disulfide bond is reduced by electrons from NADPH via FAD [10, 11]. These conformational changes are essential for enzyme function and are linked to electron transfer dynamics.

Similar to LMW‐NTRs, FFTRs form homodimers, with each protomer consisting of two structural domains: a FAD‐binding domain and a domain resembling the NADPH‐binding domain of archetypical LMW‐NTRs, which contains the redox CxxC motif. However, FFTRs differ from LMW‐NTRs in several aspects, most notably their inability to use NADPH as an electron donor and their distinctive open conformation [4, 5]. In addition, cyanobacterial FFTRs possess a unique C‐terminal tail, where the indole ring of a conserved tryptophan (W315 in *Gloeobacter violaceus*) engages in a π‐stacking interaction on the *re*‐side of the isoalloxazine ring of the FAD in the opposite protomer, potentially modulating its redox properties [4]. FFTRs also share notable structural features with NTR‐type ferredoxin‐NADP^+^ reductases, including the aromatic residue stacking onto the isoalloxazine ring [4, 12].

The catalytic model of FFTR in *G. violaceus* involves sequential ET events, starting with the binding of two Fdx1 molecules to an FFTR monomer in its open conformation. Each Fdx1 molecule donates one electron to the FAD cofactor. This mechanism entails the transient stabilization of the FAD semiquinone state (FAD_sq_) [5]. Upon reaching the FAD hydroquinone (FAD_hq_) state, a large conformational change is expected in order to bring the disulfide closer to the FAD isoalloxazine ring and thus enable disulfide reduction to a dithiol. Subsequently, the enzyme must return to an open conformation that allows the transfer of electrons to an oxidized Trx, via a dithiol–disulfide exchange reaction. This mechanism suggests that FFTR alternates between flavin‐reducing (FR) and flavin‐oxidizing (FO) conformations, akin to LMW‐NTRs [11, 13].

**Fig. 1:**
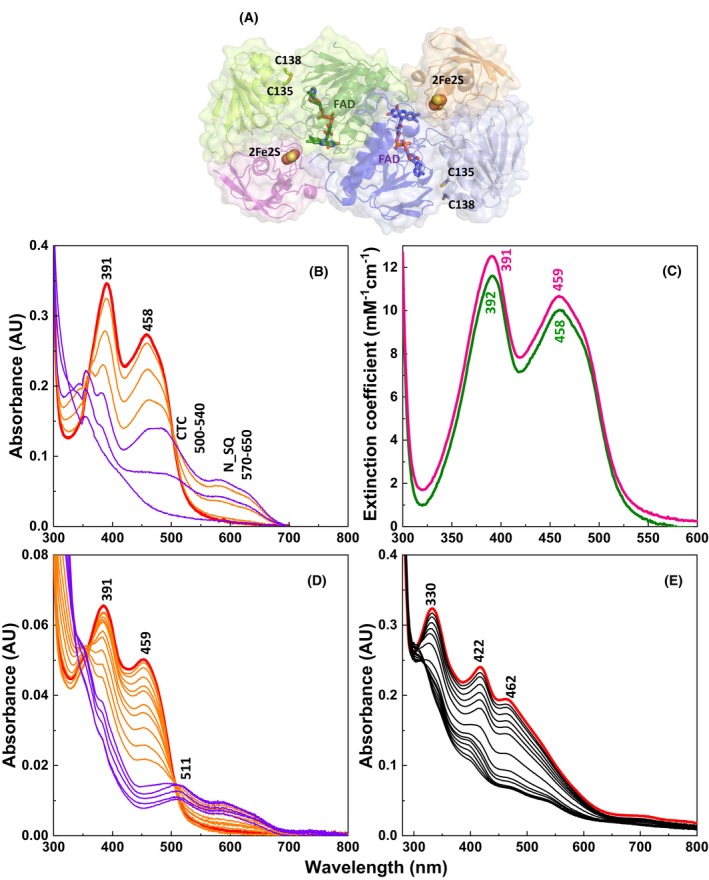
Structural and spectroscopic properties of *G. violaceus* FFTR variants and of ferredoxin 1 (Fdx1). (A) Crystal structure of the FFTR‐Fdx1 complex highlighting the redox‐active sites (PDB code 6XTF). Each FFTR protomer is represented by a blue or green ribbon diagram, with the FAD domains in dark and the redox‐active disulfide domains in light. The Fdx1 molecules are shown in pink and orange ribbons, respectively, and each protein is limited by a surface. The FAD cofactors and the Cys of the CxxC motives are shown in sticks and the [2Fe‐2S] clusters in spheres. The figure was produced using pymol (The PyMOL Molecular Graphics System, Versión 1.2r3pre, Schrödinger, LLC, New York, USA). (B) Spectral evolution during photoreduction of FFTR_WT. Flavin reduction occurs through the neutral semiquinone (N_SQ) as shown by the appearance of isosbestic points at 361 and 502 nm (for oxidized/semiquinone, ox/sq transition), and 324 nm (for semiquinone/hydroquinone, sq/hq transition) and a broad band in the 570–650 nm region. In addition, a broad feature is observed in the 450–540 nm range indicative of a thiolate‐FAD charge transfer complex (CTC) in equilibrium with the sq state. The presence of the CTC band led to an overestimation of the maximal sq stabilized in our previous report [5]. Position of absorption maxima for oxidized protein, CTC, and N_SQ are labeled in the figure. (C) Visible absorption spectra of oxidized FFTR_WT (green line) and FFTR_C135S (magenta line). (D) Spectral evolution along photoreduction of FFTR_C135S. (E) Spectral evolution during photoreduction of Fdx1. Photoreductions were performed under anaerobic conditions in the presence of 5‐deazariboflavin (4 μm) and EDTA (3 mm). Bold red spectra correspond to the initial oxidized state before photoirradiation. Spectra were recorded in 20 mm Tris/HCl, 150 mm NaCl, pH 8.0. Data are representative of three independent experiments (*n* = 3). Figure reproduced from Fig. 5.

Currently, structural data for FFTR are limited to the open FR state [4, 5]. The high‐resolution crystal structure of FFTR in complex with Fdx1 (PDB code 6XTF) shows that FFTR retains its open conformation, with FAD and disulfide redox centers still positioned far apart (Fig. 1A). The FAD remains π‐stacked onto the C‐terminal tryptophan of the opposite monomer, while the [2Fe‐2S] cluster aligns favorably with the isoalloxazine ring, suggesting efficient ET during the reductive half‐reaction [5]. However, this structure provides only limited insight into the conformational dynamics required for the oxidative half‐reaction and subsequent disulfide exchange with Trx.

To gain a deeper understanding of the enzyme's dynamic behavior, this study aimed to characterize the redox chemistry of FFTR and investigate the kinetics of its ET events using time‐resolved spectroscopic techniques, complemented with computational modeling. Our results suggest a feasible alternating mechanism within the FFTR homodimer, by means of which each subunit can assume distinct functional roles at different stages of catalysis. To mimic the dithiol‐reduced state of FFTR, we replaced one of the redox‐active cysteines in the disulfide CxxC motif with serine (SxxC) using site‐directed mutagenesis. Although this substitution had a minimal effect on the FAD midpoint reduction potential, it significantly impaired reduction by Fdx1, likely due to altered interactions between the SxxC motif and the FAD isoalloxazine ring, underscoring the critical role of this redox group in efficient ET.

Altogether, our work not only advances the understanding of the functional and structural characteristics of cyanobacterial FFTR enzymes but also provides novel insights into the processes of flavin reduction and ET reactions. Our findings reveal key differences between FFTR and archetypal LMW‐NTRs, contribute to our broader understanding of the molecular mechanisms driving redox regulation, and highlight the evolutionary diversity and biochemical adaptability of flavoproteins in various biological systems.

## Results and Discussion

### Dynamics of FFTR photoreduction and flavin redox intermediates

In a previous study, we analyzed the absorption features of wild‐type FFTR (FFTR_WT) and observed that photoirradiation induced the reduction of its FAD cofactor [5]. This process was accompanied by a decrease in absorbance at 392 and 459 nm and the transient appearance of a long‐wavelength absorbance band in the 550–700 nm region, with a maximum at 575 nm and a shoulder at 631 nm (Fig. 1B). These spectral changes indicated the stabilization of the one‐electron reduced neutral FAD_sq_, with isosbestic points at 352 and 504 nm. This behavior, commonly observed in flavoproteins, has also been reported for photoreduced *Escherichia coli* NTR in the absence of its NADPH electron donor [14]. However, in our previous analysis, we overlooked a broad band at approximately 540 nm (Fig. 1B), which we now consider to be analogous to that observed in high molecular weight NTRs (HMW‐NTRs), glutathione reductases, and lipoamide dehydrogenases [15, 16], but absent in LMW‐NTRs, the evolutionary relatives of FFTR [14]. This signal, attributed to a thiolate‐flavin charge transfer complex (CTC), has been shown to span a broad range of 450–700 nm, with a maximum around 450 nm and an isosbestic point at 503–510 nm. Thus, it partially overlaps with the neutral FAD_sq_ signal in FFTR_WT. As the photoirradiation proceeds and the flavin is further reduced, this band disappears, indicating complete reduction of the flavin. The presence of this band led to an overestimation of the maximal FAD_sq_ fraction stabilized during photoirradiation in our earlier report, where it was calculated based on the absorption at 575 nm relative to that at 450 nm. Considering the contribution of the thiolate‐flavin CTC to absorption at both wavelengths, our data indicate that the actual stabilized FAD_sq_ maximal fraction is far below 50%. This new interpretation suggests that, during photoreduction, the flavin cofactor exists in a heterogeneous distribution of redox states. These must include the oxidized forms of both FAD and disulfide, the one‐electron reduced FAD_sq_, the two‐electron reduced FAD_hq_ in FR conformation, and a distinct FAD_hq_ state adopting the FO conformation in which the disulfide‐FAD_hq_ pair evolves to a thiolate‐FAD_ox_ CTC (both EH2 states). Ultimately, the enzyme likely reaches a final thiolate‐FAD_hq_ or dithiol‐FAD_hq_ (EH4) state under the assayed photoirradiation conditions. The accumulation of FAD_sq_ and thiolate‐flavin CTC intermediates during photoreduction is not typically observed in other flavoenzymes, highlighting the unique reduction mechanism of FFTR reduction. Notably, this observation provides the first experimental evidence supporting the existence of the FO conformation in FFTR.

To further explore the factors influencing electron distribution through the flavins and the role of the disulfide bridge in modulating redox transitions, we analyzed the spectroscopic features of FFTR_C135S, a variant in which the cysteine at position 135 (Cys135) has been replaced with a serine (Ser135) so that it (partially) mimics the reduced dithiol state of the FFTR disulfide bond. The absorption spectrum of oxidized FFTR_C135S (Fig. 1C, magenta) closely resembles that of FFTR_WT (Fig. 1C, green) [5]. Stepwise photoreduction of FFTR_C135S revealed the gradual decrease in absorbance at 391 and 459 nm, which was accompanied by the appearance of a band in the 570–700 nm region, with a maximum at 589 nm, and the observation of two isosbestic points at 351 and 506 nm. Altogether, these findings suggest stabilization of a certain amount of neutral FAD_sq_ (Fig. 1D, orange bands). In common with FFTR_WT (Fig. 1B), FFTR_C135S also showed increased absorbance in the 540 nm range. However, this variant exhibited absorption features distinct from those of a thiolate‐flavin CTC, namely, the lack of the 450 nm maximum and the stabilization of a 511 nm peak during the FAD_sq/hq_ transition. Further photoirradiation failed to produce a typical FAD_hq_ spectrum, as features persisted in the 500–680 nm range (Fig. 1D, purple bands). These differences can be clearly seen when comparing the spectra shown in Fig. 1B,D. Given the irradiation periods, we suggest that these absorption features likely stem from the putative proximity of the proximal C138 thiol to FAD_hq_ and alterations in the cofactor environment caused by the C135S substitution.

When examining the behavior of oxidized Fdx1 (Fdx1_ox_), photoirradiation resulted in bleaching of its absorption maxima at 330 and 422 nm, along with a shoulder at 462 nm (Fig. 1E). These observations confirmed that Fdx1 undergoes photoreduction and formed the basis for the experiments described below. Reoxidation of all these proteins was observed upon exposure to air in the dark.

### Midpoint reduction potentials of FFTR

**Fig. 2:**
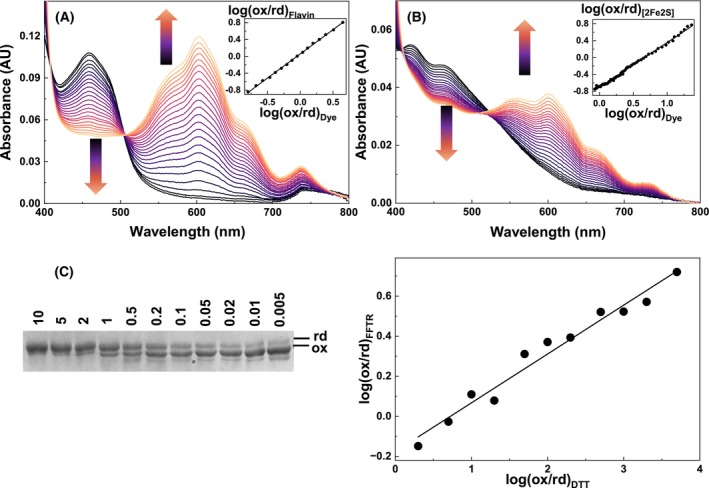
Midpoint reduction potential for FFTR_WT and ferredoxin 1 (Fdx1). Spectral evolution during the assessment of the midpoint reduction potential for (A) FFTR_WT and (B) Fdx1 in samples containing methyl viologen as dye. The visible absorption spectra, ranging from dark blue to orange, correspond to different time points during protein/dye reduction. Samples were pre‐mixed with xanthine and methyl viologen, made anaerobic by several cycles of vacuum application and bubbling with O_2_‐free argon, and subsequently mixed with xanthine oxidase. Spectra were recorded every 3–5 min for up to 2 h, at 25 °C in 20 mm Tris/HCl pH 8.0, 150 mm NaCl. Arrows represent the direction of absorbance changes at the flavin band I (~ 460 nm band), and methyl viologen (600 nm broad band). The insets show the logarithm of the ratios of oxidized (ox)/reduced (rd) dye and ox/rd protein at different time points of the reduction using absorption data at 458, 450, and 600 nm respectively for the flavin band I of FFTR, the [2Fe‐2S] of Fdx1, and the methyl viologen dye. (C) Determination of the redox potential of the disulfide in FFTR_WT. The enzyme was incubated under various redox conditions. The left panel shows separation of the rd and ox forms using non‐reducing SDS/PAGE. The [DTT_ox_]/[DTT_rd_] ratio is indicated in each lane. Band intensity was calculated using the image lab software (Bio‐rad). The right panel shows the data fitting to the Nernst equation. Data are representative of three independent experiments (*n* = 3).

We then measured the midpoint reduction potentials of FFTR_WT and FFTR_C135S to examine potential changes in the flavin properties caused by the absence of the CxxC disulfide motif. Using the xanthine/xanthine oxidase (XO) method [17, 18], we determined the FAD_ox/hq_ midpoint reduction potential, obtaining values of −448 ± 8 mV for FFTR_WT and −420 ± 8 mV for FFTR_C135S (Fig. 2A). Similarly, the midpoint potential of the FAD_ox/hq_ pair in *E. coli* NTR has also been shown to be hardly sensitive to the replacement of either of the two Cys residues of its CxxC motif with Ser [19]. We also estimated a midpoint potential of −424 ± 5 mV for the Fdx1_ox/rd_ redox couple (Fig. 2B), consistent with the requirement for a strongly reducing partner for FFTR. This indicates that FFTR is finely tuned to accept electrons from low‐potential iron sulfur proteins such as Fdx1, rather than from higher‐potential donors like NADPH (−320 mV) [20]. The midpoint potential of the redox‐active disulfide in FFTR_WT was determined to be −356 mV (Fig. 2C), positioning it between those of FAD and typical oxidized Trxs (~ −290 mV) [2]. This potential gradient supports a unidirectional and thermodynamically favorable ET pathway from Fdx1 to FAD, and subsequently to the disulfide for Trx reduction.

Altogether, these results reveal two important findings. First, despite the differences in FAD reduction observed in our experiments, the C135S substitution has little effect on its redox potential. Second, the difference in the redox potential between the FAD and the disulfide redox center of FFTR is higher than that in thioredoxin reductases [21], but follows a similar trend to those of glutathione reductase, lipoamide dehydrogenase, and HMW‐NTRs [16].

### Reduction kinetics of FFTR by dithionite

**Fig. 3:**
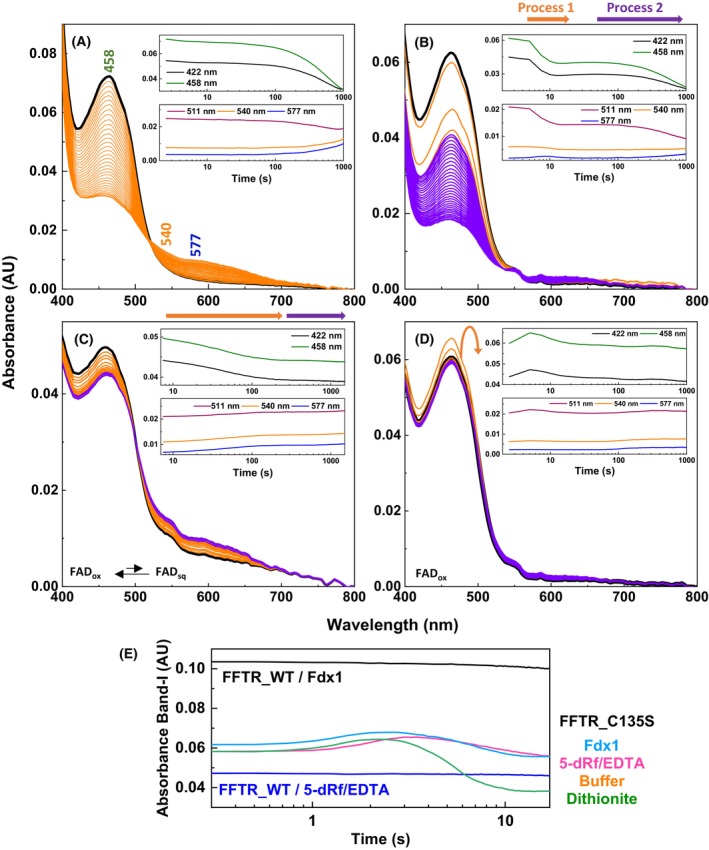
Time‐resolved spectral evolution for the FFTR reduction by non‐physiological agents. Spectral evolution observed when mixing (A) FFTR_WT (~ 3.7 μm, here after, all FFTR concentrations correspond to the homodimer) with 50 mm sodium dithionite and (B) FFTR_C135S (~ 3.1 μm) with 25 mm sodium dithionite in the stopped‐flow instrument (final concentrations after mixing are indicated). Sodium dithionite solutions were prepared in 20 mm Tris/HCl, 150 mm KCl, pH 8.0 under anaerobic conditions at 25 °C. Spectral evolution elicited by the instrument light source in (C) FFTR_WT (~ 2.5 μm) and (D) FFTR_C135S (~ 3 μm) upon mixing flavoprotein solutions with buffer solution. The rounded arrow illustrates that the absorbance initially increases and subsequently decreases. Measurements were carried out in 20 mm Tris/HCl, 150 mm KCl, pH 8.0 under anaerobic conditions at 25 °C, and contained EDTA (1.5 mm) and 5‐deazariboflavin (5‐dRf, 2 μm). In all cases, the corresponding insets portray kinetic traces at 422 nm (black) and 458 nm (green) at the top panel and 511 nm (magenta), 540 nm (orange) and 577 nm (blue) nm at the bottom panel, following maximal absorption bands for oxidized ferredoxin 1 (Fdx1), oxidized flavin, thiol or thiolate‐FAD interactions, and neutral FAD_sq_ flavin. In all panels, the first spectrum after mixing is shown in a bold black line. Traces showing absorption changes observed in the initial times upon mixing (E) FFTR_C135S (~ 3 μm) with Fdx1 (1 : 1), 5‐dRf/EDTA, buffer, and sodium dithionite and FFTR_WT with either Fdx1 (1 : 1) or with 5‐dRf/EDTA. Data are representative of three independent experiments (*n* = 3).

To investigate the ability of FFTR_WT and FFTR_C135S to chemically accept electrons, we examined the time‐absorption spectral evolution of the flavin in the visible region during reduction by sodium dithionite, a potent reductant (E° = −660 mV) that rapidly reduces flavins, particularly those with midpoint reduction potential values ranging from −200 to +100 mV [22]. For FFTR_WT, a large excess of dithionite was required to initiate flavin reduction within the stopped‐flow instrument, and even with this excess, the process was extremely slow so that complete FAD reduction was not achieved within the experimental timeframe (Fig. 3A and inset). The reduction of the flavin at 458 nm was accompanied by an increase in absorption over 500 nm, with an isosbestic point around 520 nm, which is again consistent with the coexistence of neutral FAD_sq_ and a thiolate‐flavin CTC (Fig. 3A) [15]. These data suggest a finely tuned equilibrium between these species, closely linked to conformational changes induced by ET.

In contrast, FAD reduction with dithionite in FFTR_C135S proceeded without apparent stabilization of FAD_sq_ and, as expected, of the thiolate‐flavin CTC (Fig. 3B), therefore suggesting that the C135S substitution somehow affects the redox properties of the flavin. Two main kinetic processes described the overall reduction reaction (Fig. 3B, inset). The first was significantly faster than the FAD reduction rate in FFTR_WT, while the second proceeded at a comparable rate (Fig. 3A, inset). These data indicate that the C135S replacement impacts the early stages of reduction at one of the FAD active sites, likely affecting the stability or formation of the transient FAD_sq_, without preventing the flavin from undergoing reduction. A plausible explanation for the biphasic kinetics of FFTR_C135S reduction, together with the absence of FAD_sq_ stabilization at any stage, is that the two FAD molecules within the FFTR_C135S homodimer undergo reduction to the hydroquinone state at different rates, potentially due to altered intra‐dimer interactions or asymmetric effects enhanced by the amino acid substitution.

### Kinetics of FFTR photoreduction by stopped‐flow spectroscopy

Given that both FFTR_WT and FFTR_C135S undergo photoreduction (Fig. 1B,D), we next assessed their response to the stopped‐flow light source (150 W Xenon Lamp), which served as a necessary control for subsequent experiments studying the ET reaction with the physiological donor Fdx1. Minimal photoreduction was observed without the 5‐deazariboflavin (5‐dRF)/EDTA photosensitizing system. In its presence, FFTR_WT exhibited a slight decay in flavin band I absorbance, accompanied by small increases at 540 and 577 nm, similar to dithionite reduction (Fig. 3C). Nonetheless, only a small fraction of FAD molecules was reduced, favoring the FAD_ox_ state. Over time, minor gradual increases in 550 to 650 nm and 500 to 540 nm signals were observed (inset Fig. 3C), suggesting slow FAD_sq_ and thiolate‐flavin CTC formation [15]. FFTR_C135S was even less prone to photoreduction under identical conditions (Fig. 3D). Therefore, FAD reduction was inefficient in both FFTR_WT and FFTR_C135S under these conditions.

Despite the inefficient FAD reduction, FFTR_C135S displayed a unique feature not seen in FFTR_WT: an initial slight increase in absorption at band I, followed by a small decrease (Fig. 3E, green and magenta lines in FFTR_C135S). The duration of these phases and their rates slightly varied depending on the specific mixing event measured. These findings further support the notion that the SxxC motif in FFTR_C135S influences the redox environment of the FAD cofactor, potentially altering the accessibility of reductants to the FAD active site.

### Electron transfer from reduced Fdx1 to FFTR

Building on these observations, we next explored the ET process from the reported physiological donor, Fdx1_rd_, to FFTR [5]. To do so, we analyzed the spectral and kinetic features of the reaction by anaerobically mixing photoreduced Fdx1_rd_ with either FFTR_WT or FFTR_C135S using the stopped‐flow instrument. To probe distinct stages of the ET process within the FFTR homodimer, two different FFTR : Fdx1_rd_ ratios were employed. The 1 : 1 ratio simulates a limiting condition in which only one electron equivalent is available per FFTR homodimer and would allow the observation of partial flavin reduction and the transient formation of FAD_sq_ intermediates. In contrast, the 1 : 4 condition approximates a saturating scenario that would better reflect physiological conditions, where multiple Fdx1_rd_ molecules can sequentially bind and deliver electrons to fully reduce both FAD cofactors in the homodimer. The inclusion of the FFTR_C135S variant in our analysis would serve to decouple FAD reduction from downstream ET to the disulfide.

**Fig. 4:**
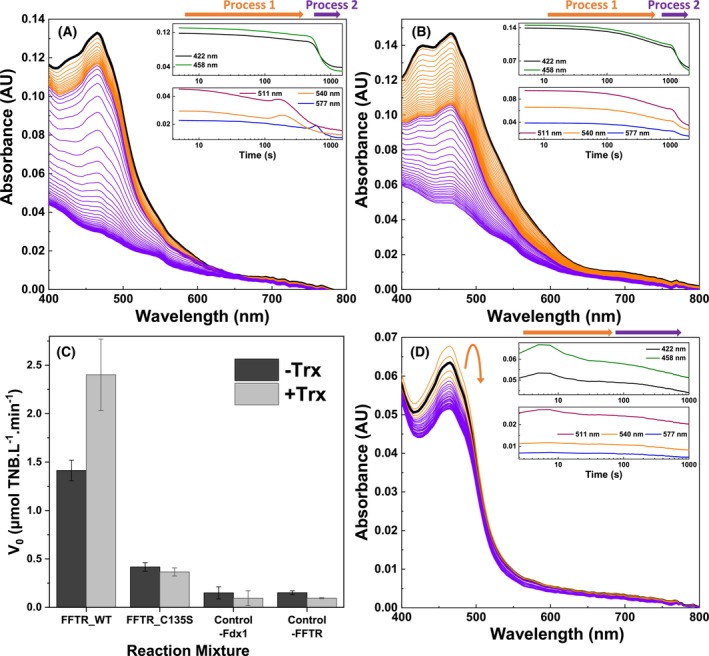
Reduction of FFTR homodimers by Fdx1_rd_ upon mixing on a stopped‐flow instrument. Spectral evolution observed upon mixing (A) reduced ferredoxin (Fdx1_rd_, ~ 5 μm) with FFTR_WT homodimer (~ 5 μm), (B) Fdx1_rd_ (~ 20 μm) with FFTR_WT homodimer (~ 5 μm) and (D) 5 μm Fdx1_rd_ (~ 5 μm) with FFTR_C135S homodimer (~ 5 μm) in the stopped‐flow instrument under anaerobic conditions. The rounded arrow in (D) illustrates that the absorbance initially increases and subsequently decreases. In all cases at least two major processes are envisaged, indicated by arrows in the panels and colored orange and purple for the first and second processes, respectively. All measurements were carried out in 20 mm Tris/HCl, 150 mm KCl, pH 8.0 under anaerobic conditions at 25 °C, and contained 1.5 mm EDTA and 2 μm 5‐deazariboflavin. In all cases, the corresponding insets portray kinetic traces at 422 nm (black), 458 nm (green) at the top and 511 nm (magenta), 540 nm (orange) and 577 nm (blue) at bottom, following respectively maximal absorption wavelengths for oxidized Fdx1, band I of oxidized flavin, thiol‐ or thiolate‐FAD interactions and neutral semiquinone FAD. In all panels the first spectrum after mixing is shown in bold black line. (C) Activity of FFTR_WT and FFTR_C135S determined by the 5,5′‐dithiobis(2‐nitrobenzoic acid) (DTNB) reduction assay using electrons from Fdx1, both in the absence (−Trx) and presence (+Trx) of thioredoxin (mean ± SD, *n* = 3). The reduction of DTNB was recorded as an increase in absorption at 412 nm and the initial velocity of the reaction was calculated. As controls, the reaction was performed in the absence of Fdx1 (−Fdx1) or FFTR (−FFTR). Data are representative of three independent experiments (*n* = 3).

Upon mixing FFTR_WT and Fdx1_rd_ at 1 : 1 and 1 : 4 ratios of their respective redox centers (FAD: [2Fe‐2S]), the spectral evolution indicated complete reduction to their FAD_hq_ states (Fig. 4A,B, respectively). This result was expected for the 1 : 4 ratio experiment but not for the 1 : 1 ratio experiment, as the amount of Fdx1_rd_ should theoretically provide only one of the two electrons required to fully reduce one of the FAD cofactors of the homodimer. This discrepancy suggests that the unavoidable photoirradiation in the mixing chamber, together with the presence of a residual 5‐dRF/EDTA photosensitizing system for Fdx1 reduction, may facilitate additional FAD reduction, either by directly reducing FAD (which seems unlikely based on the results shown in Fig. 3C) or by re‐reducing oxidized Fdx1, allowing it to transfer extra electrons. The latter seems the most plausible explanation, and it could occur without Fdx1 dissociating from the enzyme.

These observations reveal the complexity of the reduction process and highlight the importance of the experimental conditions. The spectral changes in the flavin band I region (Fig. 4A, green line in inset, at 458 nm) reflect a combination of overlapping processes, including absorption increases due to Fdx1_rd_ reoxidation (Fig. 1D) and decreases due to FAD reduction (Figs 1B and 4A). Similarly, in the neutral FAD_sq_ band region (Fig. 4A, inset at 577 nm), absorption increases may indicate the formation FAD_sq_ as well as Fdx1_rd_ reoxidation, while decreases could reflect Fdx1_ox_ reduction, the conversion of FAD_sq_ into FAD_hq_, and subsequent formation of the thiolate‐FAD CTC. This complex interplay complicates the deconvolution of the spectral evolution. Nonetheless, it is clear that the 5‐dRf/EDTA‐irradiating environment promotes flavin reduction by Fdx1_rd_, primarily leading to a FAD_hq_ state (Fig. 4A).

Furthermore, the absorption decays during the reaction correlate with FAD reduction taking place in two distinct kinetic processes, with the second process leading to the complete formation of FAD_hq_ in both monomers within the homodimer (insets Fig. 4A,B). The analysis of the time evolution of these two observed FAD reductive processes also reveals multiple overlapping events (Fig. 4A,B, insets), likely including the reoxidation of Fdx1_rd_ by FAD_ox_ to form FAD_sq_, the regeneration of Fdx1_rd_ through light irradiation, and the subsequent reoxidation of another Fdx1_rd_ to transform FAD_sq_ into FAD_hq_. The fractional accumulation and rapid conversion of FAD_sq_ to FAD_hq_ suggest that FFTR_sq_ has a higher propensity to accept an electron from the donor compared to FFTR_ox_. This behavior suggests heterogeneity in the reduction dynamics, possibly arising from a sequential ET event with homodimeric asymmetry and half‐site reactivity at any given time. The observed kinetics may reflect a stepwise reduction process in which ET within one monomer influences the redox activity and reactivity of the other (intersubunit cooperativity), leading to differences in reduction rates, transient stabilization of intermediates such as FAD_sq_, altered accessibility of the flavin cofactors within the homodimer, or alternating catalytic sites. The differing ratios of FAD and Fdx1_rd_ in the 1 : 1 and 1 : 4 experiments may also influence the dynamics of ET.

Since distinguishing between the FAD_hq_‐disulfide (EH2) and FAD_hq_‐dithiol (EH4) states is not possible in these experiments, we analyzed the feasibility of reducing the second redox‐active center, CxxC, using an enzymatic assay in which electrons transferred from Fdx1_rd_ reach 5,5′‐dithiobis(2‐nitrobenzoic acid) (DTNB) via the CxxC dithiol in FFTR. While flavoenzymes are capable of reducing DTNB in the absence of Trx, this process is considerably more efficient in its presence [23], confirming that the disulfide in FFTR is reduced by electrons from Fdx1_rd_. Furthermore, the reduction of DTNB provides evidence of ET from the dithiol in FFTR to the disulfide in Trx, corroborating the formation of the CxxC dithiol in this system (Fig. 4C). Given the continuous trend toward FAD reduction observed in the spectra (Fig. 4A,B), it is reasonable to propose that the reduction process leads to FFTR_WT in the EH4 state. The absence of detected FAD reoxidation suggests that the reduction of the flavin by Fdx1_rd_ in the reductive half‐reaction occurs faster than its oxidation by the disulfide during the oxidative half‐reaction.

Unlike FFTR_WT, the FFTR_C135S variant showed significant limitations in FAD reduction, with distinct spectral changes observed upon mixing with Fdx1_rd_ (Fig. 4D). These differences include: an initial increase in absorbance at the flavin band I after mixing, similar to other assessments of this variant (Fig. 3E), indicative of the equilibrium being shifted toward FAD_ox_ (Fig. 4D); and minimal absorption changes in the 580–600 nm region, where FAD_sq_ stabilization might occur during the process. These observations demonstrate that the C135S substitution impairs the ability of FFTR to be reduced by Fdx1_rd_. Furthermore, as expected, the FFTR_C135S variant exhibited only residual reactivity toward DTNB, likely due to the absence of a redox‐active thiol/thiolate pair (Fig. 4C).

Our findings reveal the inherent kinetic constraints of the reduction process under our experimental conditions, raising questions about its feasibility in a cellular context. The reduction process is notably slow, likely due to the highly negative redox potential of FAD within FFTR_WT, which impacts the ET from Fdx1_rd_ and limits reduction by dithionite or light. Remarkably, the observation light from the stopped‐flow apparatus was found to trigger the reduction of both the flavin in FFTR_WT and, particularly, the [2Fe‐2S] cluster of Fdx1. This light‐induced photoreduction enhances the ability of Fdx1 to transfer electrons to FFTR through a transient FAD_sq_ intermediate. Despite the possible occurrence of photoreduction artifacts in the stopped‐flow chamber under our experimental conditions, these events hardly affect the main conclusions of the study. Interestingly, a similar behavior was previously reported for the NADPH‐mediated reduction of LMW‐NTRs upon photoirradiation [14]. However, in this system, FAD_sq_ does not participate in the catalytic reaction because all its electron donors/acceptors are obligatory two‐electron exchangers. In contrast, FAD_sq_ assumes a catalytically active role in FFTRs since its electron donor, Fdx1, can only donate a single electron at a time. This requires the transient stabilization of FAD_sq_ before the binding of a second Fdx1_rd_, which transfers the second electron to generate FAD_hq_.

### Computational modeling of the FO conformation in FFTR

**Fig. 5:**
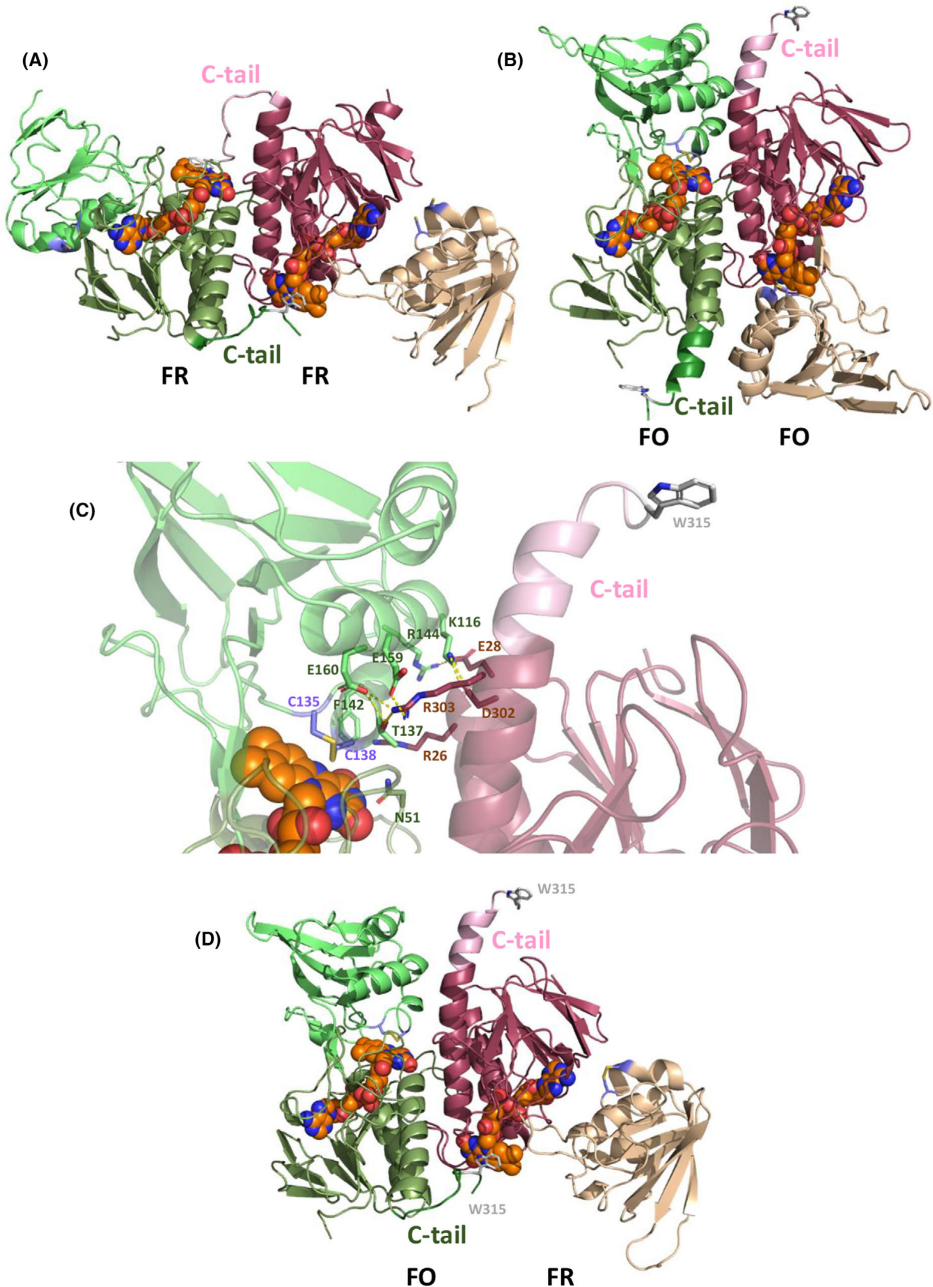
Models for the FR and FO conformations of *G. violaceous* FFTR. (A) Crystal structure of the FFTR homodimer in which both protomers are in the FR conformation (PDB code 5J60). (B) Structural model for the FFTR homodimer with both protomers in the FO conformation, as found by homology modeling using as templates the NTRs from *Mycolicibacterium smegmatis* (PDB code 5UTH) and *Mycobacterium tuberculosis* (PDB code 2A87). (C) Detail of the inter‐protomer interactions potentially contributing to stabilization of the flavin‐oxidizing (FO) conformation. Side chains of selected residues involved in dimer stabilization are shown as sticks. (D) Structural model for a putative, energy‐refined asymmetric FFTR homodimer with one protomer modeled in the flavin‐reducing (FR) conformation and the other one in the FO conformation. In all cases, protomers 1 and 2 are displayed, respectively, in green (smudge for the FAD‐binding domain, light green for the disulfide‐containing domain and forest for the C‐terminal tail) and brown (raspberry for the FAD‐binding domain, wheat for the disulfide‐containing domain and light pink for the C‐terminal tail) colors. In both protomers, the FAD prosthetic groups are shown as CPK spheres with carbon atoms colored in orange, W315 as sticks with carbon atoms in gray, and C135 and C138 as sticks with carbon atoms in violet. The figure was produced using pymol (The PyMOL Molecular Graphics System, Versión 1.2r3pre, Schrödinger, LLC).

The results presented in this work have provided the first experimental evidence supporting the existence of the FO conformation in FFTR. The architecture of FFTR, as determined by X‐ray crystallography, reveals a spatial separation of over 25 Å between the electron‐acceptor FAD molecule in one domain and the substrate's disulfide bond in the other domain (Figs 1A and 5A). Although direct structural evidence of the interaction between FAD and the disulfide is not available, the arrangement of cofactors in the crystal structure and the organization of the polypeptide chain suggest that both Fdx and S–S may interact with FAD on its *re*‐side. The formation of the thiolate‐flavin CTC described above further supports the necessary existence of these interactions. The FAD prosthetic group is securely anchored within the protein scaffold, with the only potential exposure of its isoalloxazine ring occurring through a twisting motion of the C‐terminal region of the opposite monomer within the homodimer. Additionally, the evolutionary relationship between FFTRs and LMW‐NTRs, where disulfide bonds interact with the *re*‐side of the FAD, further supports this hypothesis.

Efforts to resolve the FO conformation using crystallography have so far been unsuccessful, and computational modeling approaches based on machine learning, such as alphafold 3 [24], consistently generated structures fully superimposable with the symmetric homodimer in the FR conformation captured in the X‐ray crystal structures. Consequently, we constructed a homology model of cyanobacterial FFTR in the FO conformation (Fig. 5B). The SwissModel global quality estimates (0.7–0.8) confirm the high quality of the three‐dimensional structures of the homodimeric enzymes, with both monomers in the FO conformation (Fig. 5B) or one monomer in the FR and the other in the FO conformation (Fig. 5C,D). The latter is an asymmetric model that fully accounts for the half‐site reactivity proposed above. Moreover, both models exhibited stable structural behavior upon energy minimization and molecular dynamics simulations (data not shown), further supporting their feasibility.

In the proposed FO conformation model, the C‐terminal tail, which in the FR conformation is stabilized by π‐stacking interactions between the W315 indole ring and the isoalloxazine ring of FAD of the opposite monomer (Fig. 5A), adopts a different orientation that displaces W315 from the vicinity of FAD (Fig. 5B). As expected, the model reveals the disulfide group located close to the FAD in an arrangement comparable to that found in other LMW‐NTR family members, with the sulfur atom of Cys138 at a near‐attack distance from the isoalloxazine C4a atom and Cys135 positioned farther away from the ring (Fig. 5C). This structural arrangement suggests a potential mechanism for ET that would facilitate the reduction of the disulfide, consistent with findings in related enzymes. Thus, electrons from reduced FAD would be transferred to Cys138 to yield a thiolate anion in Cys138—likely involved in the formation of the CTC—and a thiol in Cys135.

### Catalytic mechanism and ET pathways

**Fig. 6:**
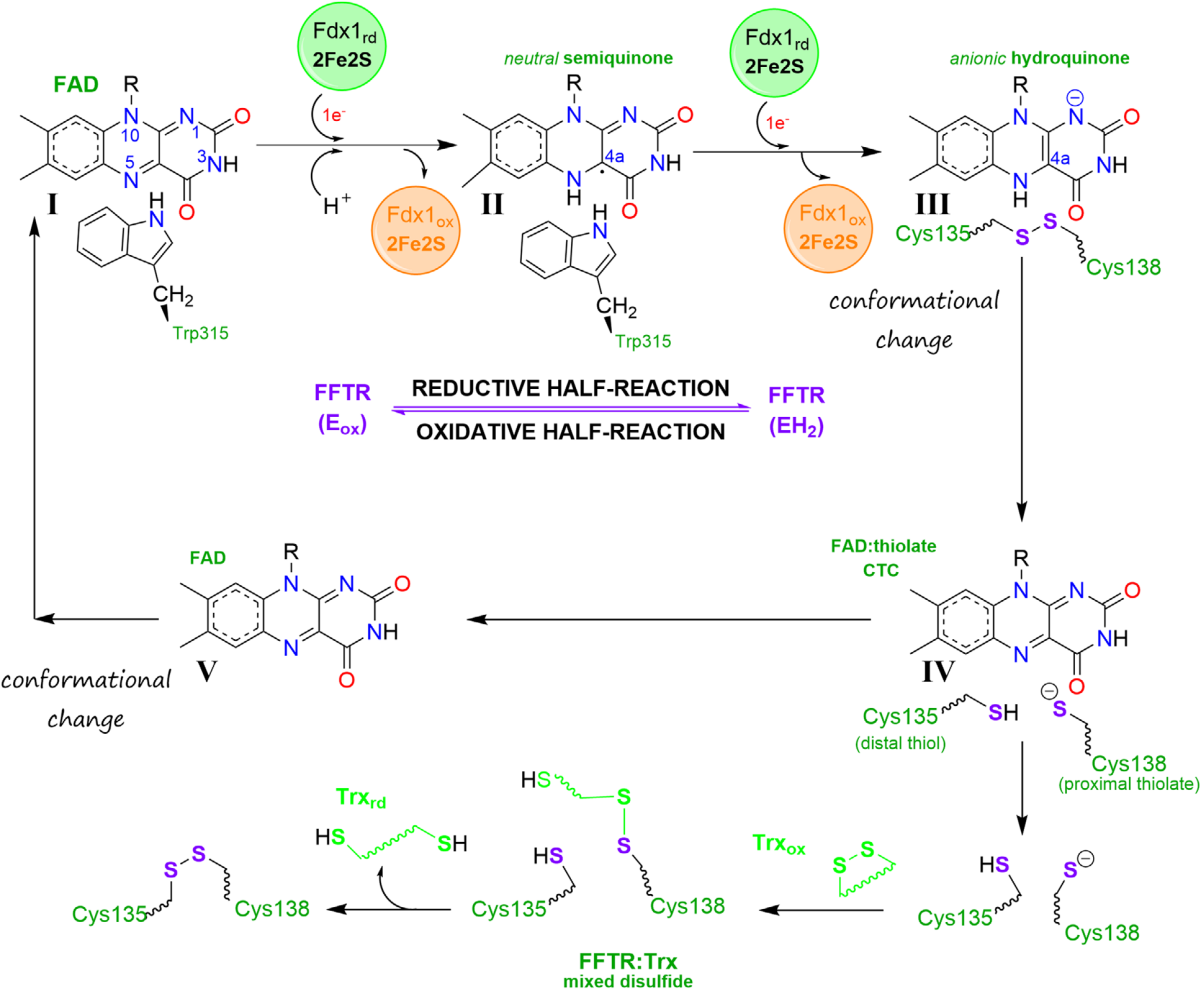
Working hypothesis for the catalytic mechanism of FFTR. During the FAD reductive half‐reaction (I and II) the FFTR maintains an open FR state that locates its oxidized FAD (FAD_ox_) proximal to the [2Fe‐2S] of reduced ferredoxin 1 (Fdx1_rd_), so as to allow electron transfer. Two Fdx1_rd_ molecules must sequentially be situated close to the FAD isoalloxazine ring of FFTR, the first one reducing it to FAD semiquinone (FAD_sq_) and the second producing the FAD hydroquinone (FAD_hq_) state. To initiate the FAD oxidative half‐reaction, a conformational change leading to a closed flavin‐oxidizing (FO) conformation (III) is required, displacing W315 in the C‐terminal tail of the twin protomer from its FAD stacking position and allowing juxtaposition of the CxxC moiety in the disulfide domain under the isoalloxazine ring of FAD_hq_. Upon disulfide bond (S–S) breakage a thiolate‐flavin charge transfer complex (CTC) is produced (IV). A subsequent intramolecular motion leading to a conformation potentially different from the initial flavin‐reducing (FR) state would then allow (i) coupling of oxidized thioredoxin to the disulfide domain and (ii) a disulfide‐dithiol exchange reaction. The eventual release of reduced thioredoxin would finally result in recovery of the starting FFTR FR state (V) and initiation of a new catalytic cycle. In the absence of Trx, a continuous supply of Fdx1_rd_ might also lead to formation of FFTR states in which both the FAD and the CxxC motif remain reduced (EH4 state).

Based on the findings presented here, we propose the catalytic mechanism outlined in Fig. 6. Our results suggest that ET from Fdx1_rd_ to Trx proceeds in a series of well‐defined steps. Initially, one Fdx1_rd_ donates an electron to an oxidized flavin that, together with a H^+^ (likely from the solvent), forms a neutral FAD_sq_ (state II in Fig. 6), which has a high affinity for accepting another electron from a second Fdx1_rd_ so that FAD_hq_ is produced (state III in Fig. 6). Although photoreduction experiments suggest that Fdx1 can be re‐reduced while still bound to FFTR, this is unlikely to occur in a cellular context. According to the structure of the protein complex (Fig. 1A, PDB code 6XTF), the [2Fe‐2S] cluster in Fdx1 is not sufficiently exposed to receive a second electron from photosystem I while bound to FFTR [5]. This implies that Fdx1_ox_ must detach from FFTR, allowing a second Fdx1_rd_ molecule to bind and complete FAD reduction, following a mechanism in which two Fdx1 molecules are required to ultimately reduce Trx, as described earlier for FTR [25].

A conformational change, involving the disruption of the interaction between the isoalloxazine of the flavin and the W315 indole ring through a swing motion of the C‐terminal tail, brings the disulfide and the FAD_hq_ together (state III in Fig. 6). At this stage, FAD_hq_ is poised to transfer reducing equivalents to the proximal disulfide. While the exact mechanism of disulfide reduction remains to be fully elucidated, our data support the formation of a flavin–thiolate CTC, indicative of intimate electronic coupling between the flavin and the cysteine residues (state IV in Fig. 6). Given these observations and mechanistic parallels in related systems [26], a hydride transfer mechanism appears plausible at this step. However, stepwise electron and proton transfer cannot be ruled out. Additional experimental evidence will be required to clarify the precise nature of this reduction step. Disruption of the flavin–thiolate CTC may result from a conformational change that alters the spatial arrangement of the reactive groups (state V in Fig. 6). In this scenario, the thiolate could subsequently engage in nucleophilic attack on the disulfide bond of Trx, thereby propagating the disulfide relay.

Kinetic data show that the reduction of the two FAD molecules within FFTR_WT occurs sequentially, following a sequential and asymmetric reduction process within the homodimer. One subunit likely drives ET, while the other facilitates structural transitions or stabilizes intermediates (Fig. 5D), indicating a coordinated mechanism may apply to related flavoenzymes and is consistent with previous findings in other homodimeric flavoenzyme systems, where the two active‐site flavins are reduced at different rates. Thus, structural evidence supports the hypothesis that the two active sites, although theoretically identical, adopt different conformational states and exhibit distinct affinities for the electron donor molecule [27, 28]. Beyond flavoenzymes, asymmetry in homodimeric enzymes is common in nature, often resulting in differential substrate affinity, allosteric regulation, or catalytic efficiency between active sites, thereby enhancing or regulating enzymatic activity [29].

Once the dithiol is formed and in the absence of Trx, two possible scenarios arise: either the enzyme stabilizes the FO conformation, awaiting Trx and potentially forming a thiolate‐FAD CTC, or the protein transitions to the FR conformation and eventually to an FR‐like conformation competent for Trx reduction, as observed in the *Clostridium* FFTR:Trx complex [6, 7]. Our *in vitro* experiments show that the slow reduction of FAD, whether by light, dithionite, or Fdx1, is influenced by the accumulation of the dithiol. During this process, a CTC is transiently formed (Figs 1C, 2A,C and 3B). However, when Fdx1 is continuously reduced, the EH4 state is slowly achieved, as evidenced by the accumulation of the FAD_hq_ form and the faster reduction of DTNB in the Trx:TR assay (Fig. 4A–C). Future studies should focus on exploring how these processes are regulated under cellular conditions.

## Conclusions

This study provides new insights into the functional mechanism of FFTR by identifying a thiolate‐FAD CTC and elucidating its redox dynamics. The highly negative FAD midpoint reduction potential of FFTR explains its preferential use of Fdx1 as an electron donor. Additionally, we demonstrate the transient nature of FAD_sq_, the sequential reduction of FADs in the homodimer, and the crucial role of the disulfide motif in flavin chemistry. Computational modeling supports an FO conformation that facilitates direct ET between the redox–active sites within the enzyme. These findings complement experimental observations, particularly those related to transient intermediates and interactions that are difficult to capture experimentally. Overall, our research advances the understanding of FFTR function and flavoenzyme biochemistry, offering new directions for future studies on redox mechanisms in biological systems.

## Materials and methods

### Protein expression and purification

FFTR_WT and Fdx1 from *G. violaceus* were prepared following the previously established methods [4]. Using the expression vector for FFTR_WT as a template, an FFTR_C135S variant was generated, following the method described in [30]. In this variant, the replacement of the CxxC redox‐active motif of FFTR_WT by SxxC prevents the intramolecular formation of a disulfide bridge. FFTR_C135S protein was prepared similarly to FFTR_WT. Briefly, the proteins were produced in Rosetta *E. coli* cells with a His‐tag at the N‐terminus along with recognition sites for either thrombin (FFTR variants) or tobacco etch virus protease (Fdx1). They were purified from the soluble fraction using Ni^2+^ affinity chromatography, and the affinity tags were removed through incubation with the respective protease. Final purification was carried out by gel filtration using a Sephacryl S‐300 HR column (Cytiva, Marlborough, MA, USA) pre‐equilibrated with 20 mm Tris/HCl pH 7.6, 150 mm NaCl.

### Spectroscopic analysis

UV–visible absorption spectra were recorded at 25 °C using a Cary 3500 spectrophotometer (Agilent Technologies, Santa Clara, CA, USA). The molar absorption coefficient for FFTR_WT (ε_458nm_ = 10.1 ± 1.3 mm ^−1^·cm^−1^) was taken from [5], the value for Fdx1 was derived from that of *Anabaena* ferredoxin (ε_423nm_ = 9.4 mm ^−1^·cm^−1^) and that for FFTR_C135S was determined herein (ε_459nm_ = 10.6 ± 0.8 mm ^−1^·cm^−1^) by thermal denaturation following previously described well‐established protocols [5, 31]. Spectral photoreductions of Fdx1 and FFTR were stepwise achieved by irradiation with a 150 W halogen lamp at room temperature for periods of up to 30 s, in the presence of 4 μm 5‐dRf and 3 mm EDTA. Photoreductions were performed under anaerobic conditions in the presence of 5‐dRf (4 μm) and EDTA (3 mm) as previously described [5, 32]. Spectra were recorded in 20 mm Tris/HCl pH 8.0, 150 mm NaCl.

### Determination of midpoint reduction potentials

Midpoint reduction potentials (*E*_m_) of FFTR variants and Fdx1 were determined using different procedures. Reduction of FFTR or Fdx1 by the xanthine/XO method [17, 18] was carried out in a closed anaerobic cuvette with a final concentration of ~ 15 μm of FFTR, 2 mm xanthine, 5 μm of the methyl viologen (*E*_m_ = −446 mV, *n* = 1), 10 mm glucose, and 10 U·mL^−1^ glucose oxidase, in 20 mm Tris/HCl pH 8.0, 150 mm NaCl. After achieving anaerobic conditions using a Schlenk line, 7.8 μg·mL^−1^ (30 nm) of bovine milk XO (Sigma‐Aldrich, Darmstadt, Germany) was added to the mixture, and spectra were recorded every 5 min for up to 2–3 h, scanning from 250 to 700 nm at 25 °C in a CARY 3500 spectrophotometer (Agilent Technologies). Absorption changes accompanying the reduction of the indicator dye and the cofactor of the flavoprotein/Fdx1 at each point during the xanthine/XO reduction were used to determine the corresponding *E*_ox/hq(rd)_ and *E*_ox/rd_ values using the Nernst equation as previously reported [18, 33].

The apparent midpoint redox potential of the disulfide bond in FFTR_WT was calculated according to [34, 35]. Redox equilibria were established by incubating FFTR at 2 μm with 0.5 mm oxidized DTT (DTT_ox_) and various concentrations of reduced DTT (DTT_rd_) for 4 h at room temperature in buffer 100 mm Tris–HCl, 2 mm EDTA, pH 8.0. Samples were resolved on non‐reducing SDS/PAGE, and the fractions of oxidized and reduced proteins were quantified using the imagelab software (Bio‐Rad, Hercules, CA, USA). The redox potential values were calculated by fitting the data to the Nernst equation.

### Kinetic measurements by stopped‐flow spectrophotometry of the FFTR FAD reductive half‐reaction

The reduction of the FAD cofactor of FFTR was evaluated by fast kinetic stopped‐flow using a SX.18MV spectrophotometer (Applied Photophysics Ltd., Leatherhead, UK) interfaced with a photodiode array (PDA) detector in the visible region according to established protocols [36]. Reduction kinetics were studied using as reductant either sodium dithionite (final concentrations in the 25–50 mm range), light irradiation from the instrument's source lamp, or the physiological electron donor in the form of photoreduced Fdx1 (Fdx1_rd_). All samples were prepared anaerobically prior to loading into the stopped‐flow system. Anaerobic conditions were achieved by several cycles of evacuation and bubbling with O_2_‐free argon. Samples were prepared in 20 mm Tris/HCl pH 8.0, 150 mm NaCl, at 25 °C, and supplemented with glucose (10 mm) and glucose oxidase (10 UI·mL^−1^) to maintain anoxic conditions. Fdx1_rd_ samples were obtained by photoreduction in the presence of 5‐dRf/EDTA (4 μm/3 mm) [36]. To follow the kinetics of photoreduction within the stopped‐flow chamber, anaerobic protein solutions, both in the absence and in the presence of 5‐dRf/EDTA (4 μm/3 mm), were mixed 1 : 1 with the same anaerobic buffer and exposed to photoirradiation from the instrument's source lamp (150 W/CR OFR Xenon Short Arc Lamp, OSRAM). Fast mixing experiments were also performed under anaerobic conditions to evaluate ET from sodium dithionite or Fdx1_rd_ to oxidized FFTR (FAD_ox_) in 20 mm Tris/HCl pH 8.0, 150 mm NaCl, at 25 °C. Absorption data at multiple wavelengths in the 360–900 nm range were collected during the reaction using the pro‐data‐sx software (App. Photo. Ltd.) [36]. Typically, 400 absorption spectra at multiple wavelengths were collected during the selected measuring times. Reduction of FFTR_ox_ by Fdx1_rd_ was studied at molar ratios of homodimer FAD_ox_ : Fdx1_rd_ of 1 : 1 and 1 : 4, with final homodimer FFTR_ox_ concentrations ranging from 2.5 to 5 μm. pro‐data‐sx and pro‐kineticist (App. Photo. Ltd.) were used to evaluate spectral evolution and observed rate constants (*k*_obs_) for the identified processes, as previously reported [36]. Nonetheless, in the specific system studied here, the deconvolution analysis proved highly complicated, likely due to multiple conformational changes and redox processes contributing simultaneously to the observed spectroscopic changes over time (see Discussion).

### Thioredoxin reductase activity assay

The Fdx1‐dependent reductase activity of FFTR from *G. violaceus* was determined by monitoring the reduction of DTNB at 412 nm, following the method described by [23]. The reaction mixture contained 0.2 mm NADPH, 0.1 μm FNR from *Anabaena* [37], 2 μm Fdx1, 0.1 μm FFTR, 0.5 μm Trx‐m, and 0.5 mm DTNB. The reactions were carried out in 100 mm potassium phosphate buffer pH 7.0 containing 2 mm EDTA at 25 °C.

### Modeling of FFTR conformations

The X‐ray structure of the FFTR homodimer with both protomers in the FR conformation (PDB code 5J60) was complemented with the structural information derived from the AlphaFold entry AF‐Q7NMP6‐F1 to generate a full‐length FR/FR molecular model. For the FO/FO structure, the NTR homodimer from *Mycobacterium tuberculosis* (PDB code 2A87) was used as a template in the SwissModel homology modeling server [23], which returned a dimer model comprising residues 7–302 in each chain. The asymmetric FR/FO FFTR model was then built by carrying out a best‐fit superposition of the FAD‐binding domain (residues 1–117 and 240–316) of chain B in the FO conformation onto the equivalent domain of chain B in the FR conformation (RMSD = 0.973 Å over 268 Ca atoms). Thus, by keeping chain A from the FR/FR dimer and chain B from its FO/FO counterpart, an initial model of the asymmetric dimer was obtained lacking only the C‐termini (303–317), which adopt different conformations depending on the redox state. To complete the model, the C‐terminal tail of chain A in the FO conformation was extended using the editing module in pymol, whereas the C‐terminal tail of chain B was grafted from chain B in PDB code 5J60 to properly position W315 in a π‐stacking interaction with the isoalloxazine ring of FAD. Energy refinement of the resulting FR/FO asymmetric model was achieved using the amber ff14SB force field [24] and the sander program from the amber 18 suite [25].

## Conflict of interest

The authors declare no conflict of interest.

## Author contributions

MB and MM conceived the project. MM‐S, VC‐P, MR, AH‐G, MM‐J, RMB, FG, MB, and MM conducted experiments and participated in data collection. MM‐J, RMB, FG, MB, and MM analyzed and interpreted the data. MB and MM wrote the paper. All authors reviewed and approved the final manuscript for publication.

## Data Availability

The model data that support the findings of this study are openly available in the ModelArchive database under the codes https://www.modelarchive.org/doi/10.5452/ma‐0nl83 and https://www.modelarchive.org/doi/10.5452/ma‐iiugx.
